# Testing the Sensory Drive Hypothesis: Geographic variation in echolocation frequencies of Geoffroy's horseshoe bat (Rhinolophidae: *Rhinolophus clivosus*)

**DOI:** 10.1371/journal.pone.0187769

**Published:** 2017-11-29

**Authors:** David S. Jacobs, Sarah Catto, Gregory L. Mutumi, Nikita Finger, Paul W. Webala

**Affiliations:** 1 University of Cape Town, Department of Biological Sciences, Rondebosch, Cape Town, South Africa; 2 Maasai Mara University, Department of Forestry and Wildlife Management, Narok, Kenya; Texas A&M University College Station, UNITED STATES

## Abstract

Geographic variation in sensory traits is usually influenced by adaptive processes because these traits are involved in crucial life-history aspects including orientation, communication, lineage recognition and mate choice. Studying this variation can therefore provide insights into lineage diversification. According to the Sensory Drive Hypothesis, lineage diversification may be driven by adaptation of sensory systems to local environments. It predicts that acoustic signals vary in association with local climatic conditions so that atmospheric attenuation is minimized and transmission of the signals maximized. To test this prediction, we investigated the influence of climatic factors (specifically relative humidity and temperature) on geographic variation in the resting frequencies of the echolocation pulses of Geoffroy’s horseshoe bat, *Rhinolophus clivosus*. If the evolution of phenotypic variation in this lineage tracks climate variation, human induced climate change may lead to decreases in detection volumes and a reduction in foraging efficiency. A complex non-linear interaction between relative humidity and temperature affects atmospheric attenuation of sound and principal components composed of these correlated variables were, therefore, used in a linear mixed effects model to assess their contribution to observed variation in resting frequencies. A principal component composed predominantly of mean annual temperature (factor loading of -0.8455) significantly explained a proportion of the variation in resting frequency across sites (*P* < 0.05). Specifically, at higher relative humidity (around 60%) prevalent across the distribution of *R*. *clivosus*, increasing temperature had a strong negative effect on resting frequency. Climatic factors thus strongly influence acoustic signal divergence in this lineage, supporting the prediction of the Sensory Drive Hypothesis. The predicted future increase in temperature due to climate change is likely to decrease the detection volume in echolocating bats and adversely impact their foraging efficiency.

## Introduction

Evolutionary biologists strive to understand the historical processes driving lineage divergence. However, the mechanisms by which lineages diverge are still among the least understood biological phenomena [1]. Nevertheless, there is a broad consensus among evolutionary biologists that, in sexually reproducing clades, new lineages arise largely by allopatric divergence [2]. When two populations become geographically isolated for long, they follow different evolutionary pathways. Typically, different geographic regions are likely to impose different selective pressures on such isolated populations due to differences in environmental conditions including climate, resources, predators and competitors. Such differences may result in the selection of divergent traits that are locally adaptive in each population. Indeed, there is evidence that geographic isolation caused by adaptation to different habitats has played a major role in lineage divergence [2].

Geographic variation manifests itself in traits such as morphology and physiology but also in behavioural ones such as acoustic signals (here used in the sense of animal sounds which carry information) [3]. Geographic variation in acoustic signals has been reported in birds [4–6], anurans [7] and bats [8–12]. Variation in sensory traits is of particular interest because these traits, whether visual, acoustic or olfactory, are involved in orientation [13, 14], communication [15], mate choice [16] and conspecific recognition [17, 18]. Divergence in these traits is therefore usually mediated by adaptive processes, rather than random processes like genetic drift [19]. This has led to the formulation of the Sensory Drive Hypothesis, which proposes that lineage diversification may be driven by environmentally-mediated differences in sensory signals [20, 21]. This hypothesis predicts a close association between geographic variation of sensory signals and environmental variables.

The function of sensory traits is dependent on the signals on which they are based (e.g. light, sound, chemicals) and transmission of these signals is in turn influenced by the local environment through which they are transmitted [19]. Geographic variation in acoustic signals, specifically, has been observed in a number of studies, many of which attributed signal divergence to current ecological conditions, providing support for the Sensory Drive Hypothesis (reviewed in Thorpe [22]). For instance, Tobias *et al*. [23] demonstrated that song divergence of bamboo-specialist birds was correlated with the sound transmission properties of their habitats, and diverged predictably across ecological gradients, suggesting that songs have become adapted to local ecological gradients.

Like bird song and frog calls, echolocation is an acoustics-based system but, unlike bird song and frog calls, it is primarily used for orientation and foraging [24, 25] rather than advertisement. However, mounting evidence suggests that social information is encoded in echolocation pulses as well [17, 18, 26–28, 29] and that echolocation may be implicated in mate choice and therefore lineage divergence [16]. In addition, echolocation is known to vary geographically over the distributional ranges of many lineages ([8, 21, 30–32]; see Jiang *et al*. 2015 [3] for a review) and this is likely due to environmental conditions. Echolocation thus provides opportunities to test the Sensory Drive Hypothesis.

Bat echolocation is influenced by several acoustic properties of the atmosphere through which it is propagated, affecting the operating range of echolocation [33]. This is due to atmospheric attenuation, which refers to the decrease in the energy of sound caused by scattering and absorption as it travels through the atmosphere [34]. Such atmospheric sound attenuation is a nonlinear function of the sound frequency, temperature, humidity and air pressure [33–35]. In general, atmospheric attenuation increases with an increase in signal frequency and relative humidity [33, 36]. Signal frequency is therefore likely to be negatively correlated with relative humidity to reduce atmospheric attenuation and optimize the operational range of echolocation. Guillén *et al*. [9], for example, demonstrated that echolocation frequencies among populations of Noack's roundleaf bat, *Hipposideros ruber* [37], in the Gulf of Guinea were inversely related with environmental humidity. However, the effect of relative humidity is mediated by a complex non-linear interaction with temperature and the interactive effects of these climatic variables [33, 35] must be considered. Such an approach was used by Mutumi *et al*. [21]. They found that, besides humidity, temperature and other climatic variables associated with latitude and altitude may play key roles in the geographic variation in bat echolocation call frequencies.

Geoffroy's horseshoe bat (*Rhinolophus clivosus* Cretzschmar, 1828) is ideal for testing the Sensory Drive Hypothesis because it has a wide geographic distribution [38] and echolocates at relatively high frequencies. This lineage, thus, presents an opportunity to test whether geographic variation in its calls exist and, if so, whether this variation can be explained by differences in climatic variables across its range. *R*. *clivosus* echolocates at relatively high frequencies (a mean of 91.7 ± 1 kHz in southern Africa; Jacobs *et al*. [39]), meaning that the impact of climatic variables on the attenuation of its echolocation calls should be relatively higher than on the calls of bats that echolocate at lower frequencies [33, 35].

Echolocation frequency of many insectivorous bats is generally known to decline as body size increases [39]. We therefore also considered the influence of body size on geographic variation in echolocation frequency. Furthermore, the call frequency of *R*. *clivosus* is much higher than expected from its body size (66 kHz) and lies outside of the 95% confidence limits of the allometric relationship between body size and call frequency for the Rhinolophidae [39]. It is possible, therefore, that climatic factors may have driven the evolution of these anomalous pulse frequencies especially because pulse frequencies of this lineage are reported to be lower in areas outside southern Africa [40].

We used a modelling approach based on Akaike’s Information Criterion (AICc) [41] to test the validity of sensory drive as an explanation for acoustic signal divergence and the anomalously high call frequencies of *R*. *clivosus*. We tested the prediction that environmental and, in particular, climatic variables are good predictors of call frequency across the distributional range of *R*. *clivosus* in eastern and southern Africa. Our objectives were to: 1) document geographic divergence, if any, in call frequency within *R*. *clivosus* and 2) assess the relative contributions of body size and environmental variables to call frequency divergence in this lineage. Our study provides insights into the climatic influence on the divergence of sensory traits and reveals potential impacts of future climatic change on faunal sensory signals.

## Materials and methods

### Ethical statement

Capture and handling of animals complied with the guidelines recommended by the American Society of Mammalogists [42], and sampling guidelines compiled by Aegerter *et al*. [43] and Kunz and Parsons [44], and were approved by the Science Faculty Animal Ethics Committee at the University of Cape Town (Clearance Number 2013/2011/V6/DJ). All workers handling bats were vaccinated against rabies and were required to use protective gloves when handling bats. *Rhinolophus clivosus* is a non-protected lineage in all countries of capture and was captured on both privately owned and protected areas under the relevant authorities as follows: Botswana (Ministry of Environment, Wildlife and Tourism, EWT 8/36/4 XVI– 78); Kenya (Kenya Wildlife Service, KWS/4001); Malawi (Department of Forestry Licence NO: 1/06/2013/1); South Africa (Northern Cape Province, Fauna 64/2010; Mupumalanga Tourism & Park Agency, MPB 5253; Cape Nature, 0035-AAA007-00081); Zimbabwe (Parks and WildlifeManagement Authority, Permit [23 (1) (C) (II) 25/2011; 19/2012 and 16/2013].

### Study animals

Geoffroy’s horseshoe bat (*R*. *clivosus*) is morphologically variable and geographically widespread with a continuous range in eastern and southern Africa, more patchy occurrences further north, and an extension into the Arabian Peninsula [38, 45–47]. We focused on the lineage distributed in eastern and southern Africa which has been shown to form a single clade distinct from populations to the north [48] The lineage exists in different habitat types across its range including deserts, savannah woodlands and fringes of forests with climates that range from arid and tropical biomes with summer rainfall areas to Mediterranean biomes with winter rainfall [38].

*R*. *clivosus* is a medium-sized bat with a mass of around 18 g [39]. It is insectivorous and forages in and around dense foliage at low heights above ground [39, 49]. *R*. *clivosus* uses high duty cycle (HDC) echolocation. The duty cycle of a periodic pulse is defined as the ratio between the duration of the pulse and its period, which is the time between the onset of successive pulses [50]. The echolocation pulses of *R*. *clivosus* pulses are typically dominated by a constant frequency (CF) component and begin and/or end with a short-frequency modulated (FM) sweep (S1 Fig; Fenton [50]; Fenton *et al*. [51]). A CF component of an echolocation pulse or tone is a narrowband signal where the sound stays constant at one frequency throughout its duration while an FM component or sweep is a broadband signal, which contains a sweep through a range of frequencies (that is frequency increases or decreases evenly within a few milliseconds).

**Fig 1:**
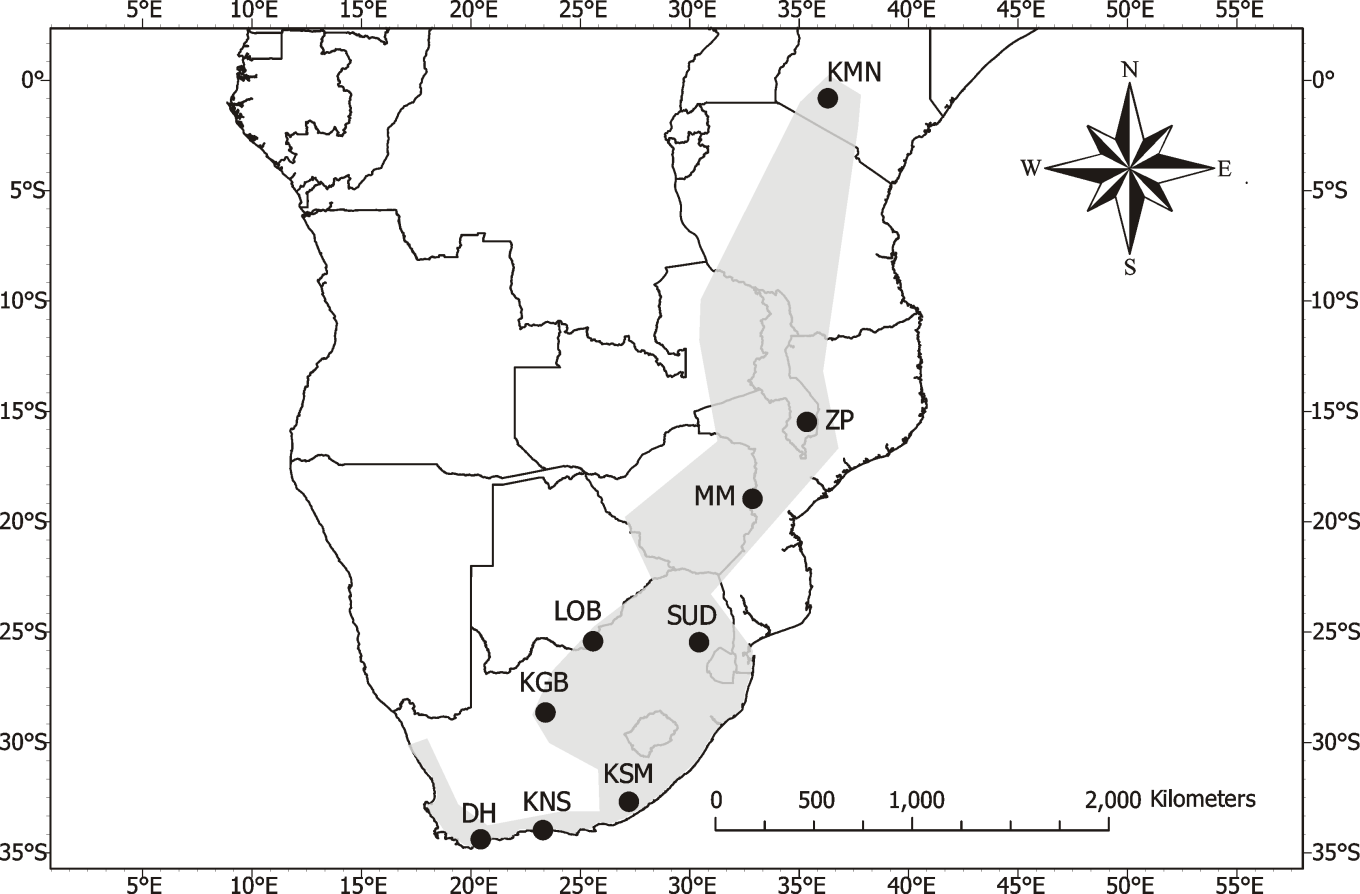
Sites across southern and eastern Africa from which *R*. *clivosus* bats were sampled. Site codes: DH = De Hoop Nature Reserve (34.42°S, 20.35°E), KMN = Kariandusi Mine (0.45°S, 36.28°E), KNS = Knysna (34.06°S, 23.22°E), KGB = Koegelbeen (28.65°S, 23.35°E), KSM = Kokstad (32.68°S, 27.19°E), LOB = Lobatse (25.24°S, 25.51°E), MM = Monaci Mine (18.88°S, 32.72°E), SUD = Sudwala (25.38°S, 30.69°E), ZP = Zomba Plateau (15.33°S, 35.28°E).

Bats were sampled from 11 different locations across the distribution of *R*. *clivosus* in Africa (Fig 1). Bats were captured at their roosts using either hand nets during the day, or mist nets at night. Sex was determined visually. We determined female reproductive condition by palpating the abdomen and inspecting the mammae [52]. Age-class was determined by examining the degree of epiphyseal–diaphyseal fusion [53]. Only adult bats were used in subsequent analyses. For ethical reasons, juveniles, pregnant or lactating bats were immediately released at the site of capture.

We chose forearm length (FA) as a proxy for body size because body mass varies seasonally and diurnally in bats (e.g. Rughetti and Toffoli [54]). FA was measured to the nearest 0.1 mm using dial callipers. Echolocation pulses were recorded from bats held 30 cm away from the microphone of an ultrasound D1000X detector (Pettersson Elektronik AB, Uppsala, Sweden - www.batsound.se) at sampling frequencies of 384 kHz and 500 kHz. We used resting frequency (*RF*), recorded from handheld bats, as opposed to frequency measurements from flying individuals to avoid the variation in pulse frequency caused by horseshoe bats compensating for Doppler shifts in frequency during flight [49]. Pulses of hand-held horseshoe bats, in contrast, have stable CF components and the inter-pulse frequency variation is low [55].

Ten to twelve pulses with the best signal to noise ratios were measured for each of 112 *R*. *clivosus* individuals (Table 1). These pulses were not chosen from the first 10 pulses of a recording because horseshoe bats are known to tune into their resting frequencies from lower frequencies after periods of silence [56, 57]. The chosen pulses were analysed using Avisoft SASLab Pro automatic measurement function (Avisoft Bioacoustics, Version 4.2, Glienicke, Germany). A Hanning window was used to eliminate the effects of background noise. The frequency at the centre of the CF component of each pulse (S1 Fig) was measured at a threshold of 18 dB below maximum. The measurements were averaged over each individual’s pulses for use in further analyses.

**Table 1:** Resting frequency, forearm length, environmental variables, and detection distances for sites sampled in this study. Means (±S.D.) and the ranges (in parentheses below the means) are given for resting frequency.

Study site	n RF Range (F)	n RF range (M)	RF RF-Range (kHz) All	Forearm length (mm)	Relative humidity (%)	Mean annual temperature (°C)	Altitude (m a.s.l)	Detection range point reflector (m)	Detection range tree (m)
**KMN**	13 99.5–100.5	1	100.06 ± 0.42 (99.50–100.60)	53.34 ± 1.21	58.41	15.3	1856.18	7.1	11.9
**LOB**	5 91.3–92.7	5 92.1–92.7	92.28 ± 0.42 (91.30–92.70)	53.88 ± 1.71	46.49	18.45	1250.68	7.6	12.9
**ZP**	1	2 79.4–81	80.80 ± 1.31 (79.40–82.00)	53.10 ± 1.18	60.39	19.95	972.62	7.3	12.3
**MM**	0	2 87.8–88.8	88.30 ± 0.71 (87.80–88.80)	54.40 ± 1.13	60.84	17.7	1221.39	7.4	12.4
**SUD**	22 89.8–92.8	15 89.6–92.5	91.46 ± 0.80 (89.60–92.80)	54.32 ± 1.40	59.12	16.68	1195.31	7.4	12.5
**DH**	15 90.4–92.2	15 91.3–92.2	91.70 ± 0.41 (91.30–92.20)	54.83 ± 1.33	63.46	16.82	137.91	7.0	11.6
**KNS**	2 90.8–92.7	5 91.7–92.7	91.86 ± 0.66 (90.80–92.70)	52.68 ± 1.08	61.29	15.98	280.06	7.3	12.2
**KGB**	4 89.8–90.1	1	89.88 ± 0.13 (89.80–90.10)	57.22 ± 1.01	43.1	16.2	1463.94	8.4	14.7
**KSM**	2 90.8–91.7	2 91.7–91.7	91.48 ± 0.45 (90.80–91.70)	53.85 ± 1.01	58.9	14.54	957.86	7.8	13.4

### Detection ranges

The distances at which bats detected a point reflector (e.g. small insect) and tree (e.g. background vegetation) were calculated for each site using Stilz and Schnitzler’s [34] online echolocation range calculator. Source levels (emitted intensity referenced to a standard distance) were originally calculated at 10 cm in front of the bats’ mouths following methods from Holderied and Helversen [58] but were adjusted to 1 meter in front of the bats’ mouths for use in the online calculator. A source level of 115 dB (n = 2 passes; unpublished data) was used across sites. This was the maximum source level measured for *R*. *clivosus* at one of our study sites (De Hoop Nature Reserve). Source levels for this lineage at the other sites are not currently available. Detection range calculations incorporated the following information: (1) climatic conditions (e.g., AMT, RH, and atmospheric pressure) at each sampled site, (2) resting frequency (Hz) of each individual bat; (3) the dynamic range, which is the difference between peak intensity (dB SPL) measured at 1 m and the auditory threshold of the bat (assumed to be 0 dB SPL for horseshoe bats [58]; (4) C1, the reflection loss which accounts for the fraction of the energy reflected, and (5) C2, the geometric spreading loss, which quantifies the loss of energy due to spreading multiplied by 2 for both outgoing emitted pulses and the returning echo. The values of the latter two factors are dependent on the geometry of the reflected wave and the web calculator therefore generates C1 and C2 depending on the target selected. Atmospheric pressure at each site was kept at that for normal atmospheric conditions, taken as 101.325 pascal.

### Environmental variables

The environmental variables used in the analyses included mean annual temperature (*AnnTemp*), relative humidity (*RH*), longitude (*Long*) and altitude (*Alt*). Altitude was identified as an important environmental variable because of the decrease in temperature (and subsequent effects on relative humidity) with increasing altitude [59]. Altitude is also a proxy for atmospheric pressure, which has a significant relationship with *RF* [21, 60, 61]. In Gillam *et al*. [61], bats used lower frequency pulses at higher altitudes and lower atmospheric pressure.

Climatic variables and altitude were derived using the geographic information system (GIS) software for analyzing geographic information, ArcGIS, following Mutumi *et al*. [21]. Average values corresponding to a 20-km radius around each sampling site were extracted. Geographic variation in any phenotypic character would take many generations to evolve and it is the conditions that many generations experience in their local habitats that influence how populations diverge. Hence, we used climatic data averaged over several years/generations (see Mutumi *et al*. [21]). Many radio-tracking studies demonstrate that medium-sized bats, like *R*. *clivosus*, forage within a radius of 10 km from their roosts (e.g., Bontadina *et al*. [62]; Goiti *et al*. [63]), and therefore we considered a 20-km zone around each roost reasonable to account for the environmental conditions to which the bats were exposed.

Sudwala (Fig 1) consisted of Sudwala and two other sites, Echo Cave (25.37°S, 30.70°E) and Elandshoek (25.38°S, 30.69°E), that were situated so close together that a 20-km radius around each of them almost entirely overlapped, meaning that the extracted averages for relative humidity and annual temperature were almost identical for these three sites. Additionally, the sites exist in the same biome [64]. Consequently, the three sites were regarded as one site centered at Sudwala for subsequent analyses.

### Statistical analyses

One-way ANOVA was used to test for differences in resting frequencies among sites, with resting frequency (*RF*) as the dependent variable and relative humidity (*RH*), mean annual temperature (*AnnTemp*), altitude (*Alt*), Latitude (*Lat*) and Longitude (*Long*) as independent variables. Data were log-transformed [ln (x + 1)] to adjust for a non-normal distribution. To account for potential multicollinearity among climatic variables, unequal variances across sites and spatial autocorrelation, we followed the statistical methods in Mutumi *et al*. [21]. Since relative humidity, annual temperature and altitude are correlated [35], we accounted for potential multicollinearity amongst these variables by extracting uncorrelated variables in the form of principal component scores using principal components analysis (PCA), as in Dormann *et al*. [65]. Furthermore, the component scores also account for the interactive effects of temperature and relative humidity [35] by combining their effects in single components. The PCA was performed on *RH*, *AnnTemp* and *Alt* in R [66] using the stats package MASS and the extracted principal component scores used in subsequent models. We tested both linear mixed effects (LME) and generalized least squares (GLS) models using the nlme package in R [67, 68]. These model structures were each fitted with different spatial autocorrelation functions to derive the best combination of model structure and spatial function to reduce autocorrelation. We used the exponential, spherical and gausian functions with and without sampling locality as a random effect (with distances defined by Latitude and Longitude). These models (including all the variables PC1, PC2, *FA*, *Lat*, *Long* and Sex) were weighted using the Akaike Information Criterion (AICc) values. The best model structure (with the lowest AICc value) was a linear mixed effects with site specified as a random effect and without any spatial autocorrelation function. This model structure sufficiently accounted for the possibility of non-normality, unequal variances (S2 Fig) and spatial auto-correlation (S3 Fig).

To test whether body size (forearm length), sex, or environmental factors (relative humidity, annual temperature and altitude) best explained the variation in *RF* across sites, a stepwise model selection by AICc was performed using the package MASS in R (http://www.R-project.org/.) and the stepAIC function. The best model was tested statistically using ANOVA to determine which predictor variables significantly contributed to the variation in *RF*.

To further explore the interactive relationship between climatic variables and RF, variables deemed significant after ANOVA on the best model were used to predict the effect of each variable on *RF* when the other variables were held constant [21]. To do so, we generated a new data set of PC scores as follows (see Mutumi *et al*. [21]). We held all except one variable constant at their mean values (calculated across all localities) and for the remaining variable we used the raw data from each locality. We then performed a PCA on these data to derive a new set of standardised PCs for each of the significant variables identified in our final best model. We ran the PC scores from this PCA in another linear mixed effects model.

## Results

### Geographic variation in resting frequencies

**Fig 2:**
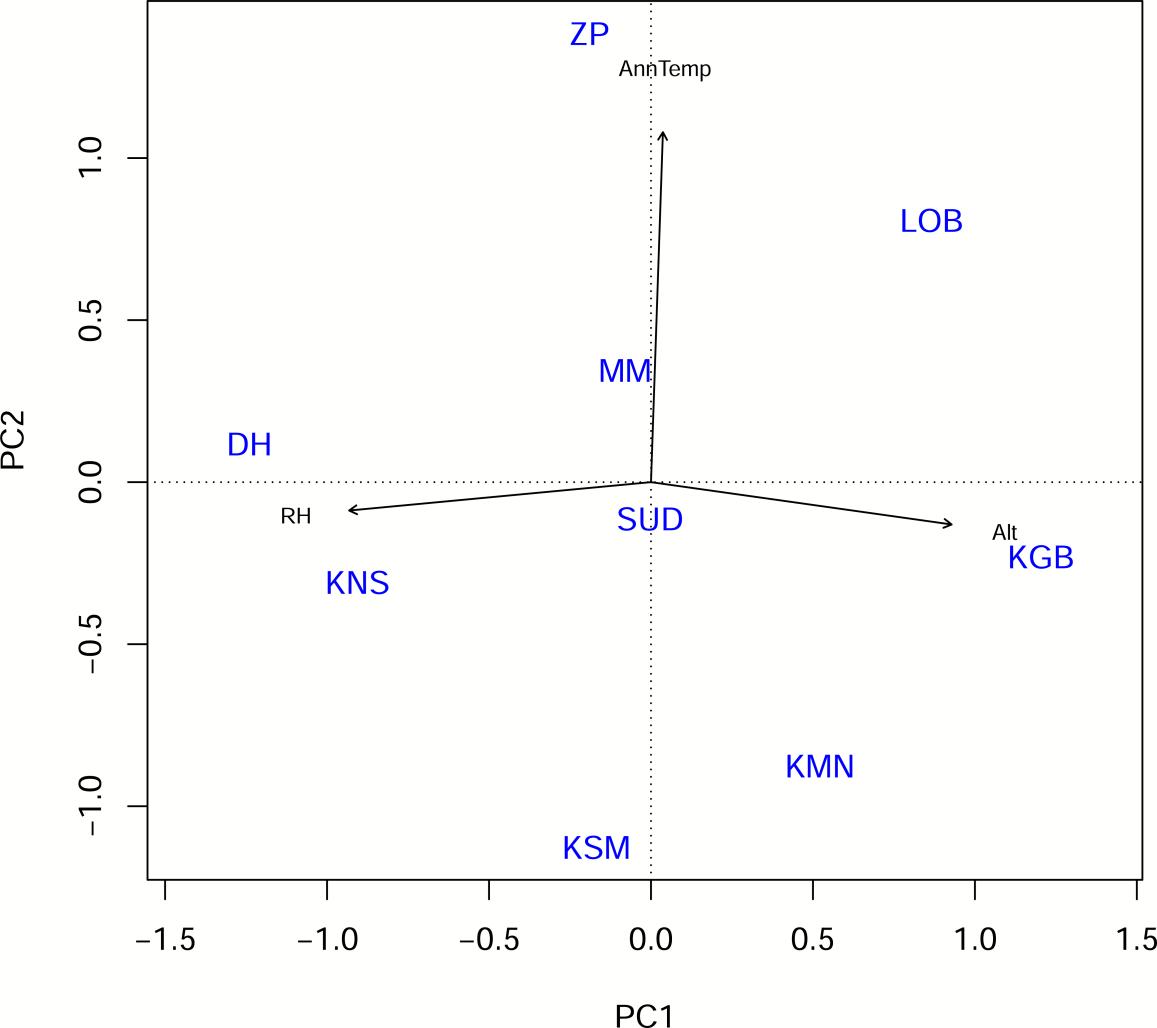
Principal components 1 and 2 extracted from PCA on relative humidity (RH) mean annual temperature (AnnTemp) and altitude (Alt). Site abbreviations are the same as in Fig 1.

The *RF* of *R*. *clivosus* varied significantly across the study sites, ranging from means of 80 to 100 kHz (*F*_8,103_ = 424.235; *P* < 0.001; Table 1). However, latitude appeared to be a weak predictor of *RF*. Although latitudinal differences between Zomba Plateau and Kariandusi Mine were smaller than between Zomba Plateau and De Hoop Nature Reserve, the difference in *RF* was greater between Zomba Plateau and Kariandusi Mine than between Zomba Plateau and De Hoop Nature Reserve (Fig 2, Table 1). Additionally, although latitude varied among South African sites, there was little variability in the resting frequencies across these sites (Fig 1; Table 1). Although our sample sizes are small for some populations, the range of echolocation frequencies within populations is low even where we had many individuals (Table 1). We therefore think that we have covered a sufficient portion of the range in RF even where we have low sample sizes.

### Principal components analysis (PCA)

The PCA yielded three principal component loadings (PC1, PC2 and PC3). PC1, on which *Alt* and *RH* loaded highest, accounted for 49% of the variation between sites while PC2, on which *AnnTemp* loaded the highest, explained 34% of the variation. In total, these two PCs therefore accounted for 83% of the variation (Table 2; Fig 2). PC3 accounted for only a small proportion of variance (17%) and was therefore excluded from further analyses.

**Table 2:** Factor loadings of each variable on each principle component derived from a PCA on climatic variables.

	PC1 (49%)	PC2 (34%)	PC3 (17%)
**Cumulative proportion**	0.489	0.826	1.000
	Variable factor loading (PC1)	Variable factor loading (PC2)	Variable factor loading (PC3)
**Relative Humidity**	0.60	0.07	0.59
**Annual Temperature**	0.02	0.64	0.09
**Altitude**	0.49	0.08	0.48

### Stepwise regression

The best model (with the lowest AICc value; Table 3) only included PC1 + PC2 + Lat + *Long* + PC1:PC2 as predictor variables. Applying ANOVA on this model showed that only PC2 was a significant predictor of *RF* across sites (Table 3). Forearm length, sex, longitude, RH and Alt (Table 2) were not therefore good predictors of *RF* across sites.

**Table 3:** The ‘best’ model from forward-backward stepwise model selection on the global model of environmental variables, body size and sex against resting frequency for *R*. *clivosus*. Statistics are only presented for variables maintained in the best model.

	numDF	denDF	ANOVA *F*-value	*P*-value
**PC1 (*RH* & *Alt*)**	1	103	0.93	0.407
**PC2 (*AnnTemp*)**	1	103	19.22	< 0.05
***Lat***	1	103	1.91	0.261
***Long***	1	103	7.53	0.071
**PC1: PC2**	1	103	4.49	0.124
**Total N = 112**				
**Number of Groups: 9**				

Abbreviations: *Alt* = Altitude; *RH* = relative humidity; *AnnTemp* = annual temperature; *Lat* = Latitude; *Long* = Longitude; numDf = numerator degrees of freedom; denDF = denominator degrees of freedom. Please note that the best model did not retain forearm length and sex.

### Predictive analysis

**Fig 3:**
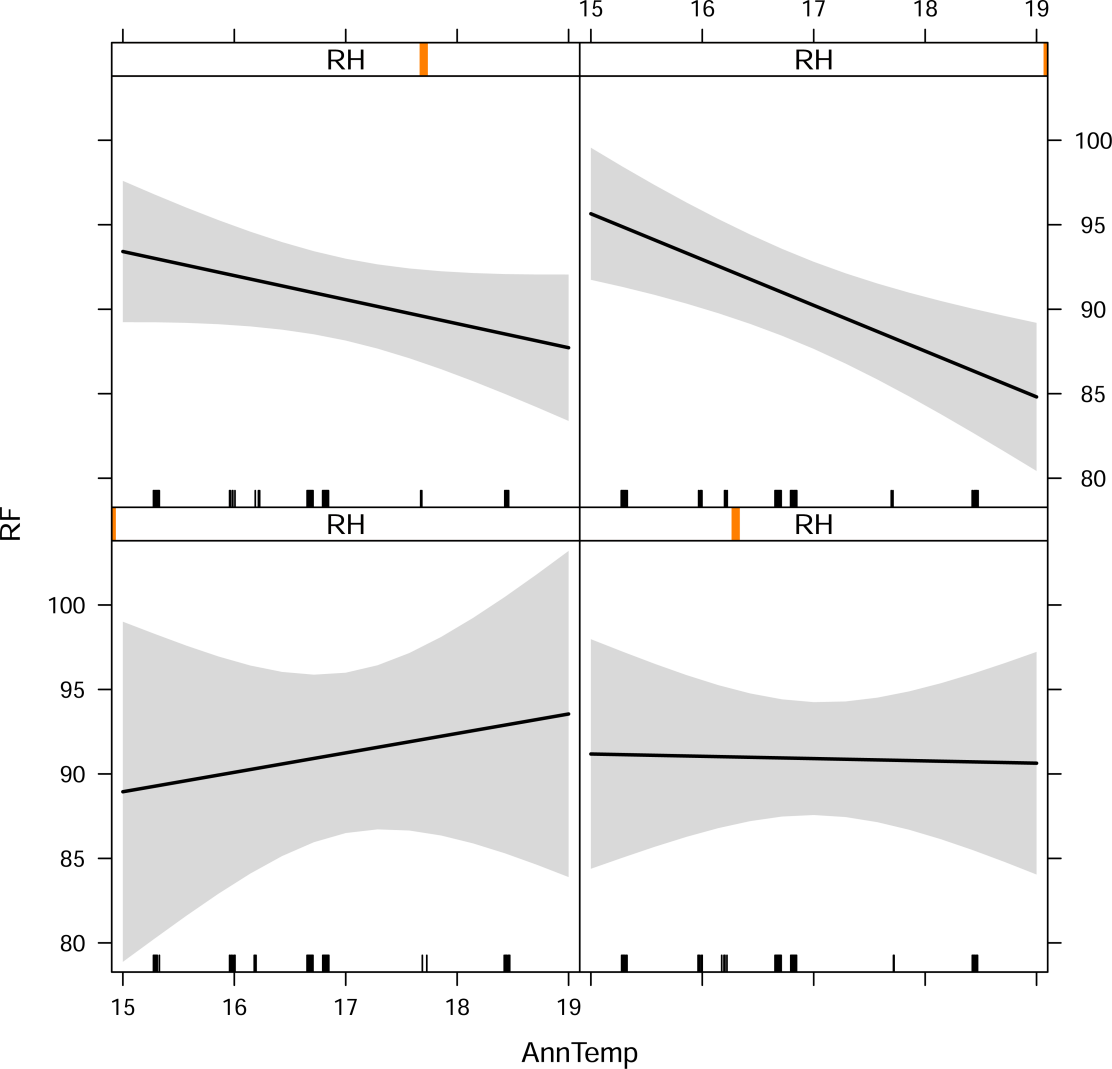
Interactive effects of relative humidity (RH; %) and mean annual temperature (*AnnTemp*; °C) on resting frequency (*RF*; kHz). Relative humidity is represented by the vertical red bars at four levels of intensity ranging from high (top left and right panels), moderate (bottom right panel) to low (bottom left plot). Details are given in the main text.

There was a complex nonlinear relationship between *RF* and two climatic variables—temperature and relative humidity (Fig 3). As the effect plots in Fig 3 demonstrate, the effect of temperature on *RF* was mediated by RH. At moderate to high relative humidity, increasing temperature led to a decrease in the *RF* used by *R*. *clivosus* (Fig 3, top two panels). At moderately low RH, there was little or no change in the *RF* (Fig 3, bottom right panel). In contrast, at extremely low relative humidity (Fig 3, bottom left panel), increasing temperature led to an increase in the *RF*s used by *R*. *clivosus*.

**Fig 4:**
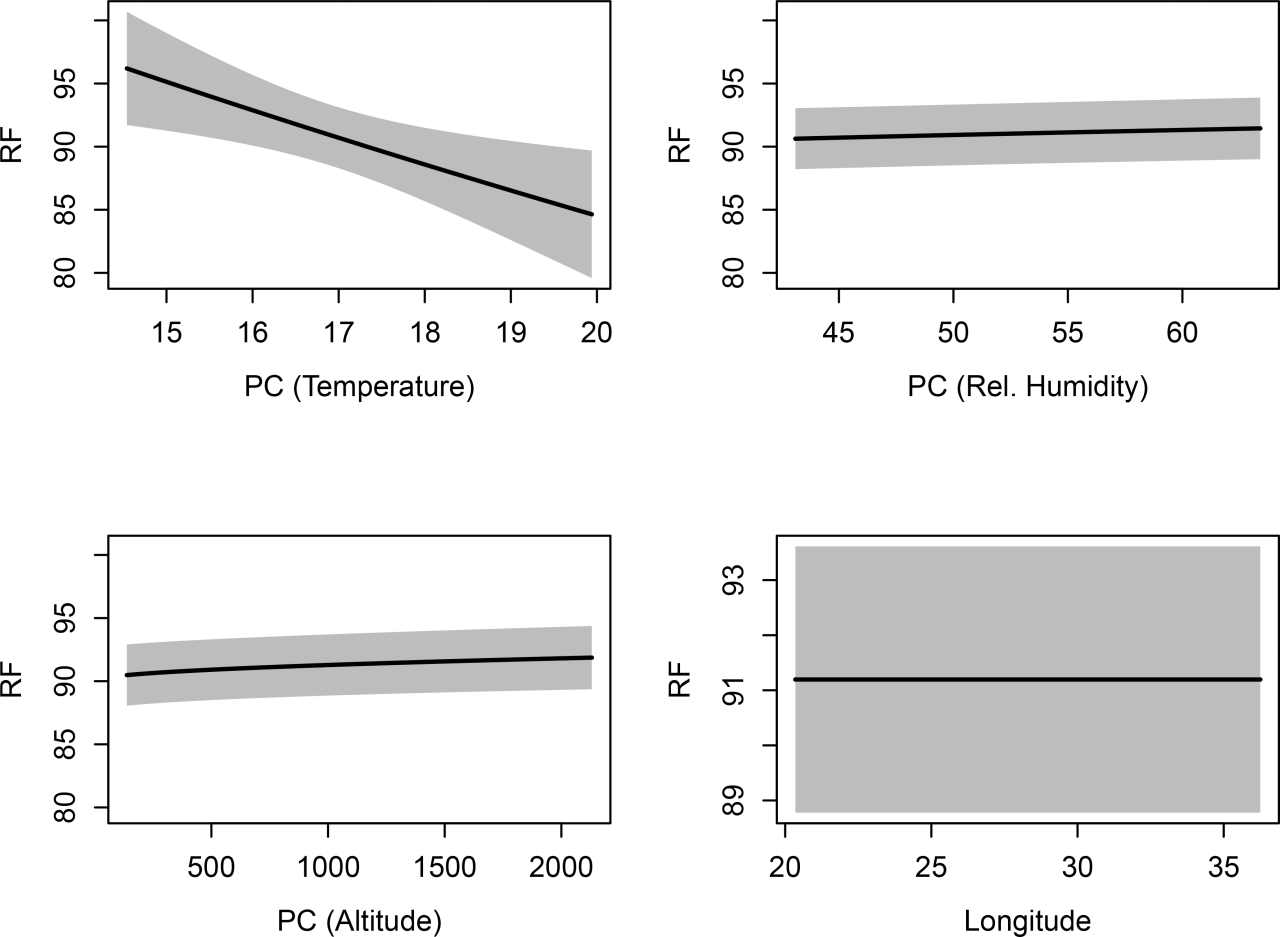
Predictive analysis plots showing how *RF* varies as each of *AnnTemp* (PC-Temperature), *RH* (PC-Rel. Humidity), *Alt* (PC-Altitude) and Longitude are varied whilst the remaining two climatic variables are kept at their mean values (calculated across study sites). Explanatory details are given in the main text.

These trends differed slightly when the effects of the climatic variables were considered together in the form of principal components (note: in this case the PCs incorporate altitude; Fig 4). The effect plots in Fig 4 revealed an inverse relationship between *RF* and temperature when *Alt* and *RH* were held constant at their mean values (Fig 4, top left panel). This trend was similar to the relationship between temperature and *RF* at high *RH* (Fig 3, two top panels). The reverse was true for the relationships between *RF* and *RH* (when temperature and altitude are held constant, Fig 4, top right panel) as well as *RF* and altitude (when temperature and RH are held constant; Fig 4, bottom left panel). A positive relationship was found when *RH*, temperature, or altitude was varied while temperature and altitude or temperature and *RH* were held constant at their mean values (Fig 4, top right and bottom left panels, respectively). There was little effect of longitude on *RF* when all climatic variables were held constant (Fig 4, bottom right).

**Fig 5:**
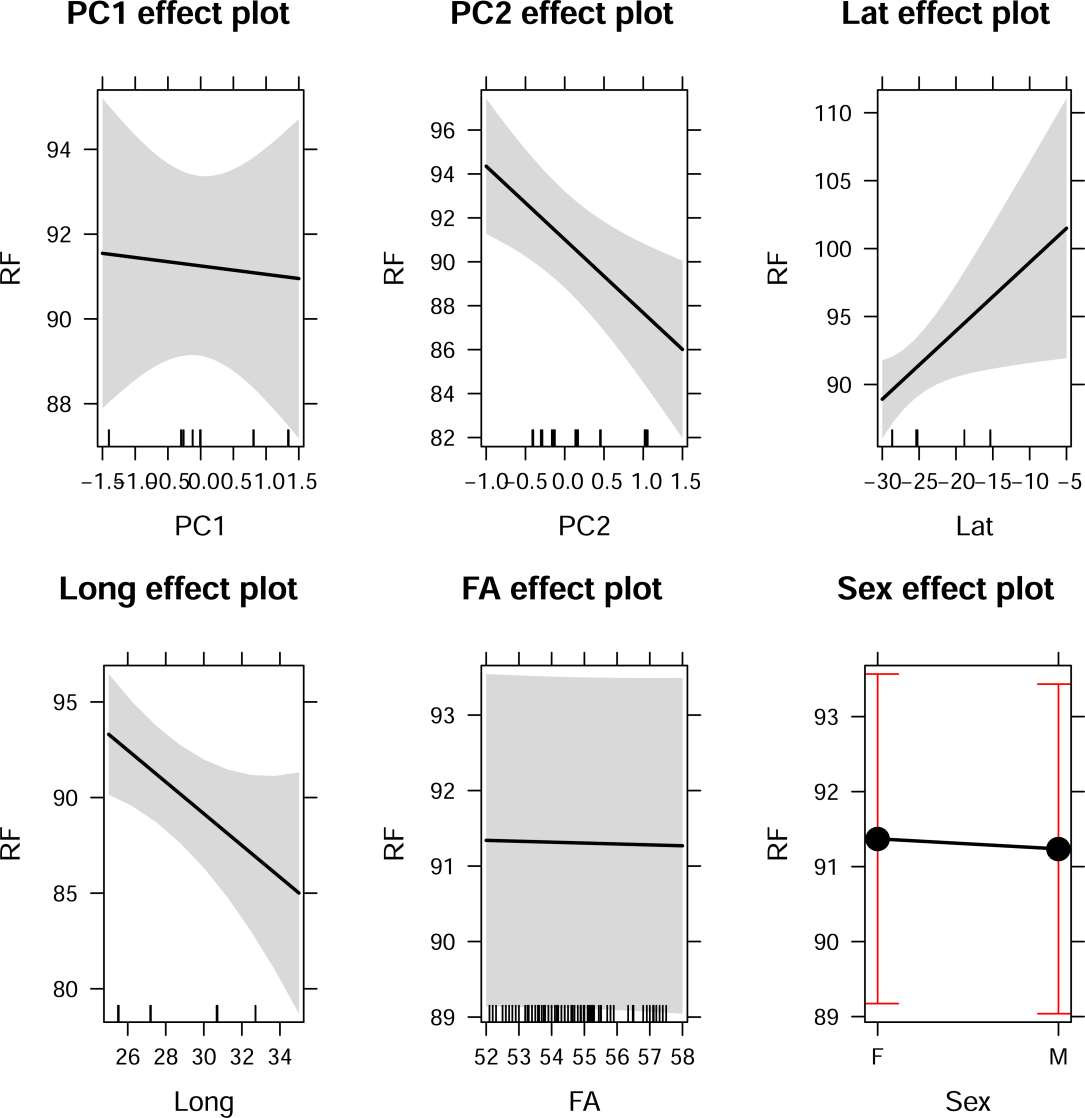
The relationship between *RF* and PC1 and PC2 in *R*. *clivosus*. **Note that the range of frequencies covered in each diagram are not the same**. Explanatory details are given in the main text.

Analyses of the individual effects of PCs showed that PC1 (RH and altitude), despite accounting for 49% of the variation in the climatic data (Table 3) had little effect on *RF* (Fig 5, top left panel). In contrast, PC2 (*AnnTemp*) which accounted for slightly less of the variation in the climatic data (34%, Table 3), strongly influenced *RF* divergence–as temperature increased, *RF* decreased (Fig 5, top center panel). There were spatial effects in both longitude (Fig 5, bottom left panel) and latitude (Fig 5, top right panel) on the divergence of *RF* pulses incorporated in the climatic effect (Fig 5). However, this spatial effect became more variable towards the equator (confidence bands became broader at lower latitudes; Fig 5, top right panel) and towards the east (confidence bands become broader; Fig 5, bottom left panel). Both of these trends may however be a sampling artefact because we had relatively few populations in the east and closer to the equator (Fig 1). Neither body size (Fig 5, bottom center panel) nor sex (Fig 5, bottom left panel) had an effect on *RF*.

### Detection distances

Detection distances varied little across sites and the difference between the maximum and minimum detection distances for a point reflector and vegetation was 1.4 m and 3.1 m, respectively (Table 1). These differences between maximum and minimum detection distances are largely due to the anomalously long detection distances in the Koegelbeen (KGB) population which is probably necessitated by the anomalously large body size of *R*. *clivosus* at this site (Table 1). Excluding this population reduces the differences to 0.7 m and 1.8 m for a point reflector and background vegetation, respectively (Table 1).

## Discussion

Our study tested the Sensory Drive Hypothesis by investigating acoustic divergence in resting frequency (*RF*) of populations of the Geoffroy's horseshoe bat (*R*. *clivosus*) across its range in eastern and southern Africa. In agreement with previous studies of other bat lineages and other taxa (e.g. Jiang *et al*. [69, 70]; Snell-Rood [36]; Sun *et al*. [71], Mutumi *et al*. [21]), we found that the variation in the resting frequencies of *R*. *clivosus* across its geographic range were predominantly climate driven, thus confirming the prediction of the Sensory Drive Hypothesis. Temperature was the best predictor of *RF* (Table 3), but its effects were mediated by RH (Fig 4). In contrast, latitude and longitude were poor predictors of *RF* (Table 3) but there were spatial effects across sites (Fig 5) suggesting that the effects of other environmental variables not considered here may be uncovered through improved sampling. Although the *RF* of *R*. *clivosus* at some localities dropped to 79 kHz, this was still higher than that expected from its body size [39]). This suggests that sensory drive cannot explain the anomalously high frequencies used by *R*. *clivosus* (see Jacobs *et al*. [39]).

The implication of our results is that the resting frequency of *R*. *clivosus* may be adapted to various temperature and relative humidity gradients across its geographic range. Although annual temperature showed the strongest effect (PC2; Fig 5), this was conditional on the degree of relative humidity at which the population occurred (Fig 3). This supports the idea that variation in *RF*s used by bats is the result of a complex interaction between pulse frequency, temperature and humidity [21, 35]. Environmental factors that impact on these three factors may thus exert an influence on the *RF*s of bats. For instance, despite being situated close to the equator, where conditions are usually warm and humid and selection should favour low pulse frequencies (high frequencies are expected to attenuate more rapidly under such conditions; [35]), bats from Kariandusi Mine in Kenya had the highest mean *RF* (100.06 kHz). However, this is probably because this site had relatively low mean annual temperature (15.30°C) and low relative humidity of 58.41% because of it having the highest altitude (1856.41 m above sea level). In support of this, Zomba Plateau with the highest mean annual temperature (19.95°C) and one of the highest values for relative humidity (60.39%), recorded the lowest frequency (80.80 kHz). This reinforces the fact that the synergistic effects of temperature, humidity and altitude on *RF* of *R*. *clivosus* had more influence than any one of the three climatic variables alone. Our study affirms earlier evidence by Mutumi *et al*. [21] in support of climate exerting a major influence on resting frequency divergence among geographically-isolated populations of two other horseshoe bats, *Rhinolophus simulator* and *R*. *swinnyi*.

Similar to the findings of Mutumi *et al*. [21], the variation in *RF* in *R*. *clivosus* also had a spatial component in that *RF* appeared to follow both a longitudinal and latitudinal cline (Fig 5). This suggests that spatial structuring of populations along the distributional range of a lineage may expose populations to additional environmental factors, besides climate (e.g. stochastic factors such as drift and/or deterministic factors such as selection under competition from conspecifics), which may also exert an influence on *RF*s.

An interesting possibility is that bats may be flexible enough to respond to temperature and RH changes over a single night and adjust their echolocation pulses accordingly. Low duty cycle bats (LDC) that do not have an acoustic fovea and do not use Doppler shift compensation [49, 55] apparently vary their echolocation frequencies by a few kHz in response to changes in temperature and RH [72]. However, apart from not being within the scope of the present study, which tries to explain geographic variation across the range of a lineage, the acoustic fovea of HDC echolocating bats and the Doppler shift compensation that their echolocation system relies upon may limit the extent to which HDC echolocating bats can adjust their pulses to deal with changes in RH and temperature. Jacobs et al. [39] found little variation in RF within individuals. Nevertheless this would be an interesting field for future research.

The geographic variation reported in *RF* in this study is not the result of the existence of cryptic lineages. Populations of *R*. *clivosus* from northern Africa and Arabia were not analysed because recent work has demonstrated that southern African populations are as genetically distinct from those further north as they are from its sister lineage, *R*. *ferrumequinum* [47]. This distinctiveness led researchers to propose a taxonomic revision across the range of *R*. *clivosus* to identify any cryptic lineages [47, 73]. Indeed, genetic analyses (as well as phenotypic characters such as forearm length) suggest that North African populations of *R*. *clivosus* may represent cryptic lineages [47] or may be divergent populations of *R*. *ferrumequinum* [47, 74]. Compared to the patchy distribution of *R*. *clivosus* in northern Africa, the eastern and southern African populations of *R*. *clivosus* form a continuous range in distribution [47], and are genetically grouped in a single clade without separation by other lineages [74]. It is therefore valid to consider the divergence in resting frequencies within the eastern and southern African populations of *R*. *clivosus* as geographic variation within the same lineage.

Contrary to the prediction of James' Rule [74] and in agreement with Mutumi *et al*. [21], *RF* did not vary with body size across the distributional range of *R*. *clivosus*, indicating that variation in body size due to climatic factors did not have an effect on *RF*. According to James’ rule, animals in cool, dry areas generally have larger body sizes than those in cool, humid areas, and that those in hot, humid areas have the smallest body sizes. If so, then it is expected that bats in hot, humid areas should have high echolocation frequencies because of the inverse relationship between body size and echolocation frequency in bats [39, 75]. However, this outcome contradicts what would be expected, considering that sound is attenuated to a greater degree in humid environments. We found no support for James’ rule, but instead our results emphasized the influence of climatic variables on echolocation frequency.

Sex was also not included in the best model (Table 3) and is therefore not a good predictor of RF variation. This result contrary to what has been reported for three other southern African rhinolophid lineages where sex was a major predictor of RF variation [21, 76]. This is probably because RF does not differ appreciably between males and females in *R*. *clivosus* (Table 1).

These results could have strong implications for insectivorous bats because of their reliance on echolocation for foraging. Frequency, in addition to source level intensity, plays a key role in the distance at which bats can detect obstacles and prey in their environment [58]. Detection ranges were surprisingly similar across sites but this is based solely on differences in pulse frequency because we used the same source level intensities across sites. It is likely that the incorporation of source level intensities for each site might yield greater variation in detection ranges. If so, then it suggests that climatic factors could affect the foraging efficiency (high ratio of prey capture to prey detection) of bats through their detection distances. The distances at which bats detect prey, especially if they use the high frequencies such as those used by *R*. *clivosus*, would be sensitive to increasing temperatures due to atmospheric attenuation of sound [35]. Luo *et al*. [35] proposed a “crossover frequency”, which is dependent on local climatic conditions, at which bats experience no change in prey detection volume in the event of increasing temperatures due to climate change. Bats that echolocate below the crossover frequency for their environment will experience an increase in their detection volume whereas bats that echolocate above the crossover frequency will experience a decrease in their detection volume as temperatures rise. For *R*. *clivosus*, this crossover frequency is at around 40 kHz [35]. This is well below the frequencies at which this lineage emits its pulses, suggesting that the detection range of *R*. *clivosus* may be sensitive to increases in temperature and relative humidity. It seems likely, therefore, that the foraging efficiency of this lineage may be negatively impacted by predicted increases in temperature as a result of global climate change. For example, if mean temperatures were to increase by only 2°C within the geographic range of *R*. *clivosus*, its prey detection volume is predicted to decrease by around 15% [35]. This could severely and negatively impact bat populations living in the tropical and sub-tropical areas where temperature is predicted to increase in the following decades. The expected warming all over Africa is very likely to be larger than the global annual mean warming throughout the continent and in all seasons, with drier subtropical regions warming more than the moister tropics [77]. These areas are part of the distributional range of *R*. *clivosus* and other Old World bats that use high duty cycle (HDC) echolocation, and therefore rising temperatures are likely to have deleterious effects on the foraging efficiency of such lineages. Unfortunately, given their reliance on an acoustic fovea and Doppler shift compensation these bats are unlikely to have sufficient flexibility to respond to rapid climatic change.

Non-adaptive explanations for acoustic signal divergence across habitats have also been proposed. If acoustic signals have a learned component, then random geographical variation is increased leading to the formation of local dialects–a form of cultural drift. This type of signal divergence has been observed in oscine passerines [23]. Although there is lack of conclusive evidence for this process occurring in rhinolophid bats, if resting frequencies are determined to some extent through a learned component and the acoustic signals of various populations are different enough to influence mate preference, gene flow between populations may be restricted, enhancing non-adaptive acoustic signal divergence [78]. However, given the strong association between climatic variables and *RF* that we report here and supported by other studies [9, 36, 69–71], it is likely that geographic variation in *RF* is the result of adaptation to local conditions rather than drift [79]. Furthermore, the anomalously high frequencies used by *R*. *clivosus* may be the result of socially mediated selection on pulse frequency for discrete frequency bands in the context of intraspecific communication [18]. If so, the evolution of high pulse frequencies in this lineage may be mediated by competition from other sympatric rhinolophid lineages for discrete frequency bands. This is a potentially fruitful area for future research.

In summary, this study provides support for the Sensory Drive Hypothesis in that *RF* varied with climatic factors. Because bats rely on acoustic signals for foraging, communication and mate acquisition, the climate-mediated divergence in these traits is likely adaptive, meaning that it could lead to lineage diversification over time, a topic in much need of further research. Further research is also needed to determine what effects rising temperatures due to climate change may have on bats, considering how their echolocation is influenced by climatic variables. Such impacts could include range contraction due to effects of increasing temperature on prey detection volume and thus bat foraging efficiency. Future studies should strive to include larger sample sizes, and possibly incorporate other environmental variables such as mean annual precipitation as well as habitat types to further tease apart the relative influence of climate on echolocation frequencies.

## Supporting information

S1 Fig A typical echolocation call for *Rhinolophus clivosus*. (TIF)

S2 Fig Residual distribution and model validation graphs for *Rhinolophus clivosus*. The top panels show the linear-mixed-effects model before correction for spatial autocorrelation. The bottom panels show the best model after all spatial autocorrelation structures with and without study sites as a random effect have been tested.
(TIF)

S3 Fig Spline correlograms of the residuals (with 95% confidence intervals) from a linear mixed effects model with study sites as random effects, including all predictor variables. (TIF)
