# Supplementary figures and images for: Testing the Sensory Drive Hypothesis: Geographic variation in echolocation frequencies of Geoffroy's horseshoe bat (Rhinolophidae: Rhinolophus clivosus)
Source: PLoS One. 2017 Nov 29;12(11):e0187769. doi: 10.1371/journal.pone.0187769 (PMC5706677; doi:10.1371/journal.pone.0187769)

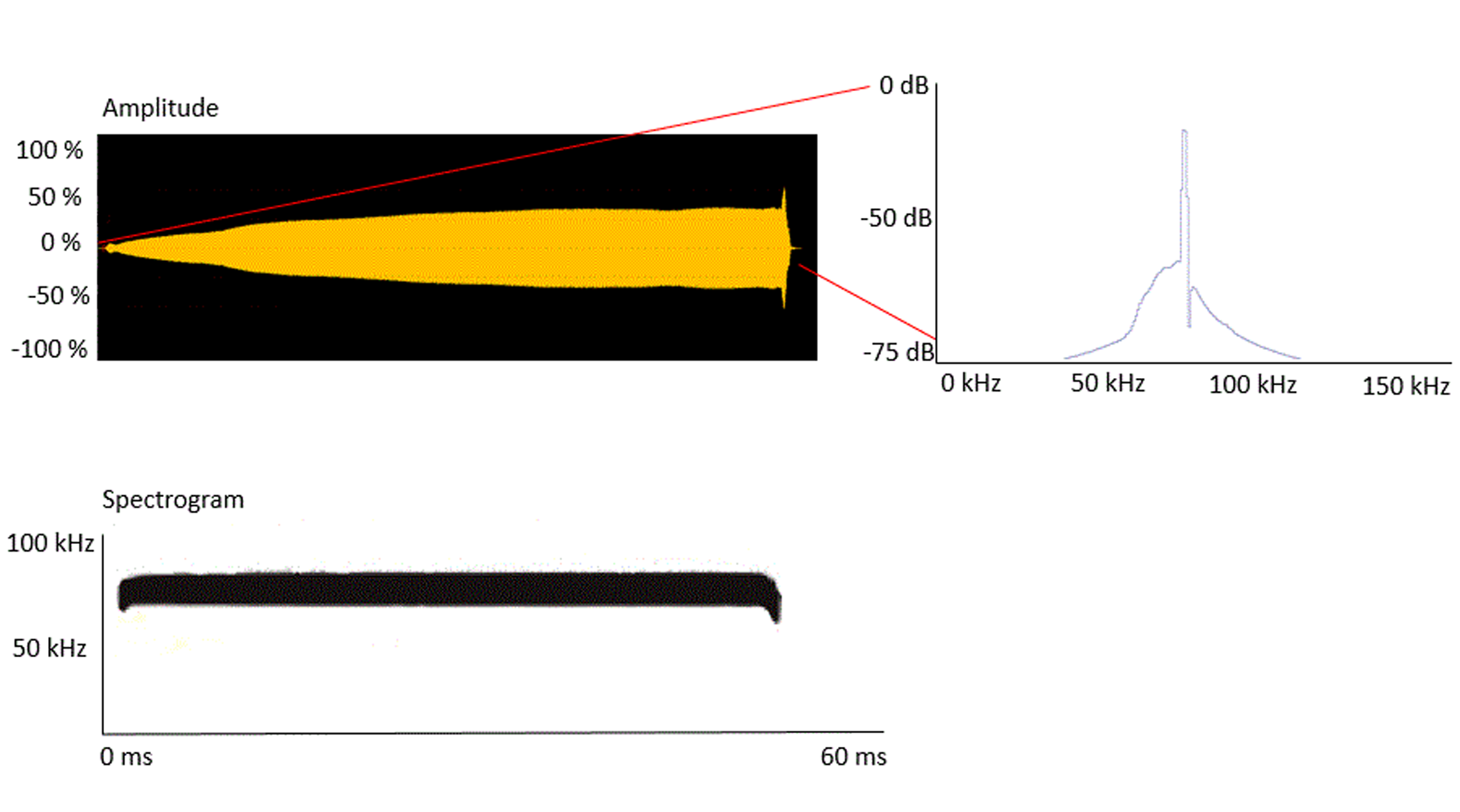

Supplement: S1 Fig — (TIF) [file pone.0187769.s001.tif]

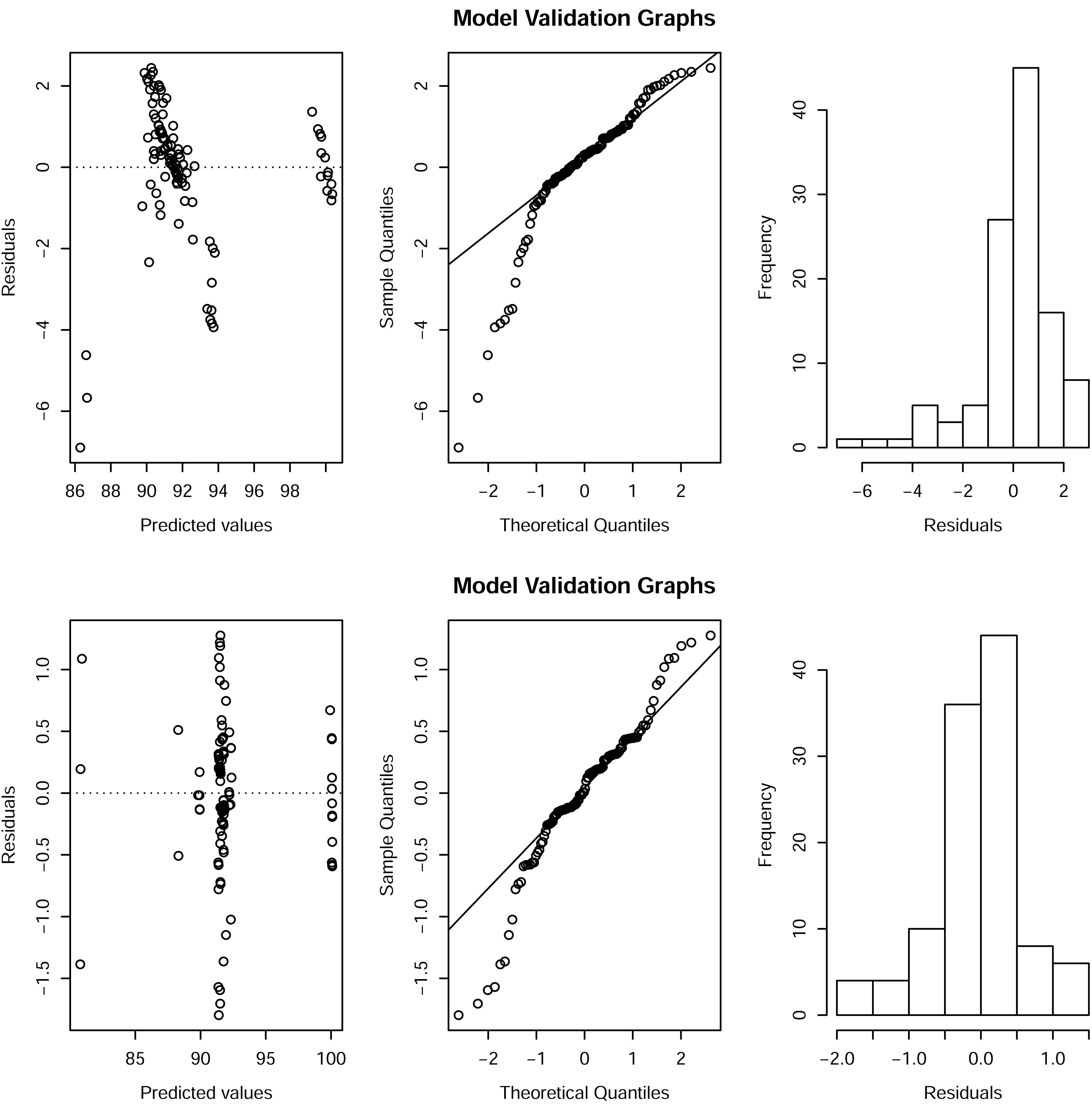

Supplement: S2 Fig — The top panels show the linear-mixed-effects model before correction for spatial autocorrelation. The bottom panels show the best model after all spatial autocorrelation structures with and without study sites as a random effect have been tested. (TIF) [file pone.0187769.s002.tif]

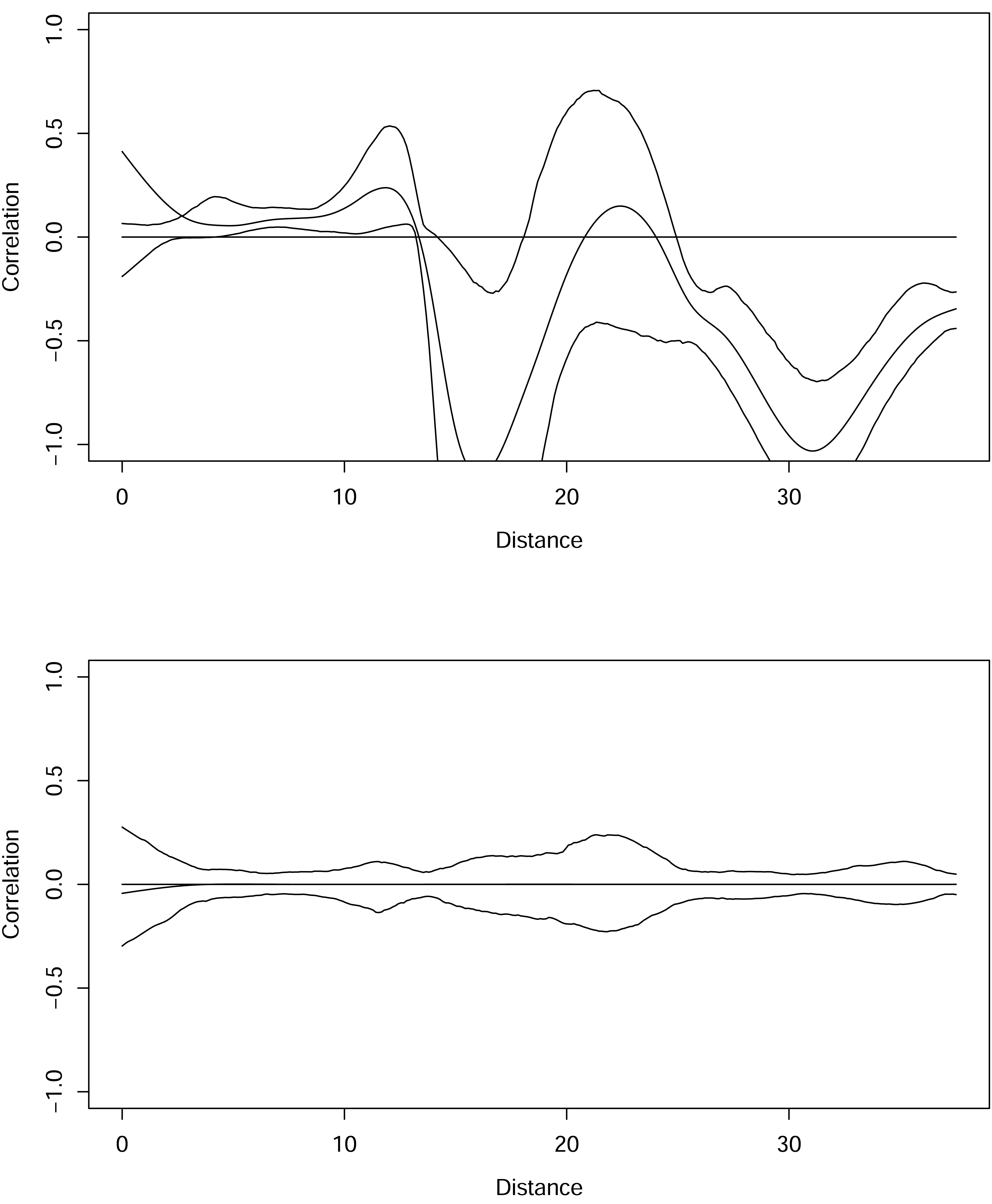

Supplement: S3 Fig — (TIF) [file pone.0187769.s003.tif]
